# Micro-RNA Profiling of Exosomes from Marrow-Derived Mesenchymal Stromal Cells in Patients with Acute Myeloid Leukemia: Implications in Leukemogenesis

**DOI:** 10.1007/s12015-017-9762-0

**Published:** 2017-09-16

**Authors:** Juliana Barrera-Ramirez, Jessie R. Lavoie, Harinad B. Maganti, William L. Stanford, Caryn Ito, Mitchell Sabloff, Marjorie Brand, Michael Rosu-Myles, Yevgeniya Le, David S. Allan

**Affiliations:** 1 Regenerative Medicine Program, Ottawa Hospital Research Institute, Ottawa, ON Canada; 2 Centre for Biologics Evaluation, Biologics Genetic and Therapies Directorate, Health Products Food Branch, Health Canada, Ottawa, ON Canada; 3 Ottawa Institute of Systems Biology, Ottawa, ON Canada; 4 Department of Biochemistry, Microbiology and Immunology, University of Ottawa, Ottawa, ON Canada; 5 Department of Cellular and Molecular Medicine, University of Ottawa, Ottawa, ON Canada; 6 Hematology, Department of Medicine, The Ottawa Hospital and University of Ottawa, Ottawa, ON Canada; 7 Radiobiology and Health, Canadian Nuclear Laboratories, Chalk River, ON Canada; 8 Ottawa Hospital Research Institute, 501 Smyth Rd, Box 704, Ottawa, ON K1H 8L6 Canada

**Keywords:** Mesenchymal stromal cells, Acute myeloid leukemia, MicroRNA, Exosome, Bone marrow

## Abstract

Gene regulatory networks in AML may be influenced by microRNAs (miRs) contained in exosomes derived from bone marrow mesenchymal stromal cells (MSCs). We sequenced miRs from exosomes isolated from marrow-derived MSCs from patients with AML (*n* = 3) and from healthy controls (*n* = 3; not age-matched). Known targets of mIRs that were significantly different in AML-derived MSC exosomes compared to controls were identified. Of the five candidate miRs identified by differential packaging in exosomes, only miR-26a-5p and miR-101-3p were significantly increased in AML-derived samples while miR-23b-5p, miR-339-3p and miR-425-5p were significantly decreased. Validation of the predicted change in gene expression of the potential targets was investigated by interrogating gene expression levels from public datasets of marrow-derived CD34-selected cells from patients with AML (*n* = 69) and healthy donors (*n* = 40). Two molecules with decreased gene expression in AML (EZH2 and GSK3β) were predicted by the miR profiling and have been previously implicated in AML while three molecules were increased in AML-derived cells and have not been previously associated with leukemogenesis (KRBA2, RRBP1 and HIST2H 2BE). In summary, profiling miRs in exosomes from AML-derived MSCs allowed us to identify candidate miRs with potential relevance in AML that could yield new insights regarding leukemogenesis or new treatment strategies.

**Electronic supplementary material:**

The online version of this article (10.1007/s12015-017-9762-0) contains supplementary material, which is available to authorized users.

## Introduction

Perturbations in the bone marrow microenvironment in acute myeloid leukemia (AML) favor the selective progression of leukemia over normal hematopoiesis [1]. Although leukemia cells may contain intrinsic mechanisms for chemo-resistance and disease progression, changes in gene regulatory networks induced by extrinsic signals from the tumour microenvironment have been demonstrated in recent preclinical animal models [2, 3]. Moreover, many patients with AML relapse months or years after initial treatment, suggesting leukemic cells remain quiescent within protected niches until they re-emerge as chemorefractory disease [4]. The occurrence of donor derived leukemia following bone marrow transplantation also supports the concept of a damaged microenvironment that is permissive to leukemia development [5]. Signals arising within the microenvironment may be central to leukemic progression by altering gene regulatory networks within leukemic cells that enhance their competitive advantage at the expense of normal progenitors. Identifying specific signalling pathways that may be implicated and defining the origin of signals within the marrow microenvironment have proven challenging.

Mesenchymal stromal cells (MSCs) are central to the integrity of the bone marrow microenvironment as they give rise to all cell types of the marrow stroma, including adipocytes, osteocytes and fibroblasts [6]. Recent studies [7, 8] have described marked changes in MSC function in patients with AML compared to healthy controls. In addition to altered growth kinetics and differentiation potential, MSCs from patients with AML have altered expression of genes associated with the induction of quiescence in hematopoietic progenitors [7]. MSC-derived signalling within the bone marrow may explain the origins of marrow-derived changes observed in AML that support leukemic dominance over normal hematopoiesis.

Exosomes are microvesicles formed from intracellular endosomes in MSCs and other cell types. Microvesicles encapsulate signaling molecules such as receptors, chemokines, mRNA and microRNA (miR) [9]. Bioactive signals within exosomes can mediate cell–cell signaling following exosomal release into the extracellular milieu and are implicated in altered tumour microenvironments, including leukemia and other hematological malignancies [10, 11]. Exosomes can merge with target cells and release signalling molecules that can alter gene regulatory pathways through epigenetic or non-epigenetic mechanisms, profoundly altering gene expression and the phenotype and behaviour of tumor or normal cells. The extent to which MSC-derived exosomal contents are altered in AML is not known and the role of MSC-derived exosomal signalling in AML has not been established. In this report, we profiled the miR contents of MSC-derived exosomes from patients with AML and controls to identify miRs that may be implicated in leukemic progression. Our interest in miRs relates to their known capacity to influence signaling pathways and previous work that highlighted the role of deleting the miR processing gene Dicer-2 in osteoblasts which can induce leukemia in mice [12]. By comparing gene expression levels of potential targets of specific miRs in leukemic cells compared with normal hematopoietic progenitors, we have identified novel regulatory pathways implicated in MSC-derived exosomal miR signalling in AML.

## Methods

### Human Bone Marrow Samples and Mesenchymal Stromal Cells (MSCs)

Cells were obtained from filters following normal bone marrow harvests and bone marrow aspirates from newly diagnosed AML patients, in accordance with the Ottawa Health Sciences Network Research Ethics Board (see Table 1), and were characterized as previously reported [8]. Briefly, bone marrow mononuclear cells (MNCs) were prepared using Ficoll-Paque Plus density gradient as per manufacturer’s instructions (GE Healthcare, Pittsburg, PA, USA), as recently described in our previous work [7, 8]. MNCs were seeded in complete Dulbecco’s Modified Eagle Medium (DMEM, Gibco/Life Technologies, Carlsbad, CA, USA) supplemented with 10% fetal bovine serum (FBS, Hyclone/Thermo Scientific, Waltham, MA, USA) and 1% penicillin/streptomycin (Multicell/Wisent Inc., St. Bruno, QC, CA), at a density of 1.0–1.5 × 10^7^ cells per T-75 flask. The emergence of plastic-adherent MSCs was followed for 3–6 weeks in a humidified tissue culture incubator (5% CO_2_, 21% O_2_, 37 °C) with fresh medium changes performed every 7–10 days. MSCs were characterized by flow cytometry, in accordance with criteria established by the International Society for Cellular Therapy (ISCT), [13] using the following panel of antibodies: FITC-CD14, APC-CD19, PE-CD73, PE-CD90, APC- CD105, PE-Cy7-CD146 (BD Biosciences, Mississauga, ON, CA); PE-Cy7-CD34, FITC-CD45 (eBioscience, San Diego, CA, USA). MSC differentiation potential was assessed using Human MSC Functional Identification Kit (R&D Systems, Minneapolis, MN, USA) in accordance with manufacturer’s instructions and as previously described [7, 8].

**Table 1:** Characteristics of patients and healthy controls recruited in the study

Subject	Age	Gender	Diagnosis
1	51	F	AML (NOS), complex cytogenetics
2	64	M	AML (M5), normal cytogenetics
3	68	M	AML (NOS), hypodiploidy
4	46	M	Healthy control
5	40	M	Healthy control
6	24	M	Healthy control

### Exosome Isolation and RNA Sequencing

Passage 4 MSCs were expanded to 90% confluency (as described above) in complete growth medium containing exosome-free FBS (SBI, Palo Alto, CA, USA). Exosome-free medium was used to ensure that no contaminating exosomes were present from bovine serum. Conditioned medium (CM, 10 ml) was obtained after 48 h from confluent cultures and kept frozen at − 80 °C. Exosome extraction of CM was performed by SBI Biosciences using ExoQuick-TC reagent and miRNA extraction using SeraMir kit (SBI, Palo Alto, CA, USA). Quality and quantity of obtained RNA was verified using Agilent Bioanalyzer Small RNA Kit prior to generation of libraries using NEBNext Multiplex Small RNA Kit (New England Biolabs, Ipswich, MA, USA). Next generation sequencing was performed using Illumina HiSeq 2500 platform with 100 bp paired end runs (Illumina, San Diego, CA, USA). Sequencing data for all samples was normalized to picograms of miR.

### Bioinformatic Analysis

Putative candidate miRs were selected as either those that: 1) were significantly more or less abundant (difference in means, *p* < 0.05) or 2) had a relative level of more than 1.5-fold difference between AML- and control-derived MSC exosomes (*p* < 0.05). Interaction networks were built using Ingenuity Pathway Analysis (IPA) (version 24390178) which queries the Ingenuity Pathway Knowledge Base for interactions between each candidate miR and all molecules stored in the Knowledge Base. Only findings related to the human species were considered. We further inquired about the effect of the candidate miRs on the direction of change in expression of the connected genes within the network using the Molecule Activity Predictor (MAP) tool. This in silico approach enabled us to visualise the overall effect of the candidate miRs on their interactome.

We next enquired whether the prediction models derived from the in silico analyses performed using IPA could be validated with the Gene Expression Omnibus (GEO) database repository at the National Center for Biotechnology Information (NCBI). Using the GEO2R interactive web tool (NCBI) that allows the identification of genes that are differentially expressed from two groups of samples, we analyzed GEO datasets of AML samples and CD34-selected healthy controls (cut-off criteria for gene selection was log_2_(fold change) of ± 1.5 in gene expression with adjusted p-value of < 0.05).

Levels of known targets, or interacting partners (interactome), derived from 3 GEO datasets (GSE30029, GSE12662 and GSE17054) were then determined from AML samples and CD34-selected healthy controls. These data sets were identified through a search of GEO datasets containing AML samples with concomitant healthy controls using systematic gene expression analysis.

### RT-qPCR Analysis of CD34-Selected Cells from Bone Marrow

Mononuclear cells were isolated from bone marrow aspirates of patients with AML and healthy controls as described above. Lineage-negative (Lin^−^) BM cells were obtained using the EasySep™ Human Progenitor Cell Enrichment Kit with Platelet Depletion to enrich for stem and progenitor cells (HSPCs). Lin^−^ cells were directly stained for CD34^+^ (Clone 4H11) cell surface marker and sorted for CD34^+^ cells using the Beckman Coulter MoFlo sorter. RNA was isolated (Arcturus PicoPure Kit, LifeTech) from Lin^−^CD34^+^ cells and rDNase treated (Qiagen). RNA was converted to cDNA using SuperScript II (Invitrogen), according to manufacturer’s instructions. cDNA was used for qPCR using primers listed in Supplemental Table 1. qPCR was done using LC480 (Roche).

## Results

### Profiling miRs in MSC-Derived Exosomes

**Fig. 1:**
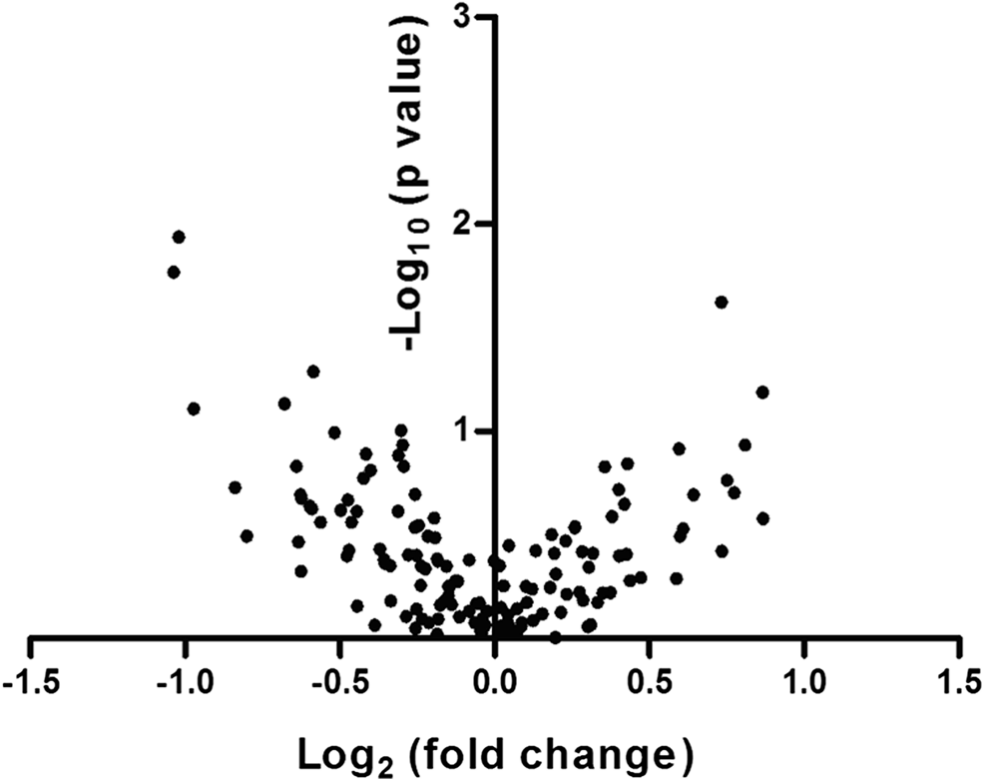
Volcano Plot of microRNA isolated from exosomes of AML-derived MSCs and control MSCs. The log_2_ of microRNA levels in AML-derived samples in comparison to healthy control samples (fold-change) is plotted against the –log_10_ of the p value in a Student’s t test after comparing the mean fold change. A total of 3 microRNA species were significantly reduced (more than 1.5-fold reduced or –log_2_(0.6) and *p* < 0.05 or –log_10_(1.3)) in AML-derived samples compared with controls and 2 microRNA species were significantly increased. The specific microRNA species are presented in Table 2

MSCs used in these experiments to derive exosomes were previously characterized and reported [8]. The identity and copy number (normalized per pg microRNA) of 259 microRNAs was determined for each sample. A total of 19 miR species were not present in any of the AML-derived MSC exosomes, while all 259 were detected in at least one of the control samples. The identity and copy number of the 30 most abundant miRs were not different between AML-derived samples and healthy controls (see Supplemental Table 2). Differential packaging of exosomal microRNAs between AML-derived samples and controls was investigated by comparing mean levels of each microRNA species (per pg of RNA) and mean fold-differences in miR levels compared with healthy controls (see Fig. 1). MicroRNAs with significant differences in copies per pg RNA and/or fold differences compared with healthy controls were identified and are presented in Table 2. Of the five candidate microRNA species that were differentially packaged, only miR-26a-5p and miR-101-3p were significantly increased in AML-derived samples while miR-23b-5p, miR-339-3p and miR-425-5p were significantly decreased in AML-derived samples compared to controls. The five candidate microRNAs were not differentially expressed in the parental MSCs following analysis of genome-wide gene expression data that was previously published using these same cells (data available in public dataset) [8], indicating that the differential abundance of these miRs is strictly due to differential exosomal packaging.

**Table 2:** miRs from exosomes derived from normal MSCs and AML-derived MSCs that are significantly different based on comparison of mean copy number (Student’s t test, *p* < 0.05) and/or the unadjusted fold-change was significantly different (*p* < 0.05)

Micro RNA	Mean copy number per pg microRNA, AML MSC exosome vs control	Fold change in AML exosome vs control
miR-26a-5p	14.4 ± 3.3 vs 7.3 ± 1.2, *p* = 0.024	1.6-fold change, *p* = 0.07
miR-101-3p	36 ± 11.6 vs 17 ± 6.7, *p* = 0.065	1.8-fold, *p* = 0.02
miR-23b-5p	0.38 ± 0.66 vs 5.3 ± 1.8, *p* = 0.011	0.49-fold change, *p* = 0.028
miR-339-3p	0.0 ± 0 vs 3.7 ± 1.6, *p* = 0.017	0.49-fold change, *p* = 0.02
miR-425-5p	0.57 ± 0.99 vs 5.9 ± 3.8, *p* = 0.07	0.51 fold change, *p* = 0.037

### Gene Expression of miR Targets in AML-Derived and Healthy Donor CD34-Selected Cells

Known targets of the five candidate miRs were identified as described in the “Methods” section. In addition, the predicted change in gene expression of the target interacting partner was assessed based on whether the candidate miR was increased or decreased in AML-derived samples compared to controls. The known targets and their predicted change in expression are presented in Tables 3, 4, 5, and 6. There are no known predicted targets of miR-23b-5p. Proteins, microRNAs and/or other molecules that regulate the candidate miRs were not included in our analysis and are not presented in the tables.

**Table 3:** Effect of increased miR-101-3p levels, as detected in AML-derived MSC exosomes, and validation of gene expression levels (log_2_ fold change of AML/control of ± 1.50 and adjusted p value of < 0.05 are reported) from datasets GEO-GSE30029, GEO-GSE12662 and GEO-GSE17054

Target	Effect of miR	Predicted change	Target function	GEO-GSE30029	GEO-GSE12662	GEO-GSE17054
LIN28B	Inhibits, D	↓	Other	NS	NS	NS
ATM	Inhibits, I	↓	Phosphatase	NS	NS	NS
SUZ12	Inhibits, I	↓	Enzyme	NS	NS	NS
EZH2	Inhibits, I	↓	Transcription regulator	− 1.61, *p* < 0.0001	NS	NS
RNF2	Inhibits, I	↓	Transcription regulator	NS	NS	NS
PPKDC	Inhibits, I	↓	Phosphatase	NS	NS	NS
Akt	Inhibits, I	↓	Complex/group	NS	NS	NS
BMI1	Inhibits, I	↓	Transcription regulator	NS	NS	NS

EZH2 is decreased in AML, as predicted, in GEO-GSE30029

*D* Direct inhibitor, *I* Indirect inhibitor

**Table 4:** Effect of increased miR-26a-5p levels, as detected in AML-derived MSC exosomes, and validation of gene expression levels (log_2_ fold change of AML/control of ± 1.50 and adjusted p value of < 0.05 are reported) in AML-derived cells vs controls from datasets GEO-GSE30029, GEO-GSE12662 and GEO-GSE17054

Target	Effect of miR	Predicted change	Target Function	GEO-GSE30029	GEO-GSE12662	GEO-GSE17054
GSK3B	Inhibits, I	↓	Phosphatase	NS	NS	− 1.60, *p* = 0.002
PGR	Inhibits, I	↓	Nuclear receptor	NS	NS	NS
EPHA2	Inhibits, I	↓	Phosphatase	NS	NS	NS
PTEN	Inhibits, I	↓	Phosphatase	NS	NS	NS

GSK3B expression changed in same direction of predicted change in GEO-GSE17054

*I* Indirect inhibitor

**Table 5:** Effect of decreased miR-339-3p levels, as detected in AML-derived MSC exosomes, and validation of gene expression levels (log_2_ fold change of AML/control of ± 1.50 and adjusted p value of < 0.05 are reported) in AML-derived cells vs controls from datasets GEO-GSE30029, GEO-GSE12662 and GEO-GSE17054

Target	Effect of MiR	Predicted change	Target function	GEO-GSE30029	GEO-GSE12662	GEO-GSE17054
KRBA2	Inhibits, D	↑	Other	2.32, *p* < 0.0001	NS	NS
RRBP1	Inhibits, D	↑	Other	1.96, *p* < 0.0001	NS	NS
CST4	Inhibits, D	↑	Other	NS	NS	NS
CASC10	Inhibits, D	↑	Other	NS	NS	NS
FAM169B	Inhibits, D	↑	Other	NS	NS	NS
ZNF747	Inhibits, D	↑	Other	NS	NS	NS
LOC100506422	Inhibits, D	↑	Other	NS	NS	NS
HIST2H2BE	Inhibits, D	↑	Other	2.34, *p* < 0.0001	NS	NS
CST1	Inhibits, D	↑	Other	NS	NS	NS
C19orf35	Inhibits, D	↑	Other	NS	NS	NS
PPP1R12B	Inhibits, D	↑	Phosphatase	NS	NS	NS
PRCD	Inhibits, D	↑	Other	NS	NS	NS
CENPBD1	Inhibits, D	↑	Other	NS	NS	NS
CECR1	Inhibits, D	↑	Enzyme	NS	NS	NS

KRBA2, RRBP1, and HIST2H2BE changed in same direction as predicted in GEO-GSE30029

*D* Direct inhibitor

**Table 6:** Effect of decreased miR-425-5p levels, as detected in AML-derived MSC exosomes, and validation of gene expression levels (log_2_ fold change of AML/control of ± 1.50 and adjusted p value of < 0.05 are reported) in AML-derived cells vs controls from datasets GEO-GSE30029, GEO-GSE12662 and GEO-GSE17054

Target	Effect of miR	Predicted change	Target function	GEO-GSE30029	GEO-GSE12662	GEO-GSE17054
RFPL4B	Inhibits, D	↑	Other	NS	–	–
DYNC1I2	Inhibits, D	↑	Other	NS	NS	NS
SPATA6L	Inhibits, D	↑	Other	NS	NS	NS
LPA	Inhibits, D	↑	Other	NS	NS	NS
PLN	Inhibits, D	↑	Transporter	NS	NS	NS
ZNF844	Inhibits, D	↑	Other	–	NS	NS
SSX3	Inhibits, D	↑	Other	NS	NS	NS
HNRNPA3	Inhibits, D	↑	Other	NS	NS	NS
ZNF286B	Inhibits, D	↑	Other	–	–	–
IFITM1	Inhibits, D	↑	Transmembrane receptor	NS	NS	NS
ZNF695	Inhibits, D	↑	Other	NS	–	–
KIR3DS1	Inhibits, D	↑	Other	–	NS	NS
EYS	Inhibits, D	↑	Other	–	NS	NS
BCL2L2-PABPN1	Inhibits, D	↑	Other	–	–	–
ARL17A/B	Inhibits, D	↑	Other	NS	NS	NS
KRTAP4-1	Inhibits, D	↑	Other	NS	NS	NS
APOBEC3A	Inhibits, D	↑	Enzyme	− 2.33, *p* = 0.008	NS	NS
CALM1	Inhibits, D	↑	Other	NS	NS	NS
ACAN	Inhibits, D	↑	Other	NS	NS	NS
TBC1D29	Inhibits, D	↑	Other	NS	NS	NS
PCMT1	Inhibits, D	↑	Enzyme	NS	NS	NS
S100A7A	Inhibits, D	↑	Other	NS	NS	NS
TMEM155	Inhibits, D	↑	Other	NS	NS	NS

APOBEC3A was not changed in the same direction as predicted in GEO-GSE30029

*D* Direct inhibitor

Validation of the predicted change in expression of the target genes was investigated by interrogating gene expression datasets of marrow-derived CD34-selected cells from patients with AML and controls identified through a systematic search of the GEO repository. Three datasets were identified and gene expression data were analyzed for each of the known targets in AML-derived samples (total from all 3 datasets, *n* = 69) and healthy donor CD34-selected cells (total from all 3 datasets, *n* = 40). The characteristics of the three GEO datasets are summarized in Table 7. All three datasets used array-based gene expression profiling of CD34-selected cells from bone marrow of patients with AML or healthy controls. The number of molecules in each dataset that demonstrated significant differences in gene expression between AML-derived samples and controls depended on the sample size with a range of 606–1728 mapped molecules with significant differential expression. Of particular note, one dataset only included patients with the M3 subtype of AML (GEO-GSE12662) and none of the predicted changes in target gene expression by the candidate miRs identified in our study (no cases of AML-M3) were validated in this dataset.

**Table 7:** Gene expression datasets identified in systematic search

GEO datasets	Array	AML samples (bone marrow)	Controls (bone marrow)	Molecules, IPA-mapped	Molecules in dataset
GSE30029	Illumina Human HT-12 V3.0	CD34+ (*n* = 46)	CD34+ (*n* = 31)	33,471	1728
GSE12662	Affymetrix Human Genome U133 plus 2.0	CD34+ (AML M3; *n* = 14)	CD34+ (*n* = 5)	44,622	1316
GSE17054	Affymetrix Human Genome U133 plus 2.0	CD34+ (*n* = 9)	CD34+ (*n* = 4)	44,622	606

Criteria for inclusion in the dataset used for analysis: Adjusted p-value cut off: <0.05 and Log_2_ FC (AML/control) cut-off: ± 1.5

Increased miR-101-3p was predicted to inhibit the expression of 8 potential targets (1 directly and 7 indirectly). Only reduced expression of EZH2 was validated in GEO-GSE30029 with a 0.33-fold change in expression relative to controls (log_2_ value of − 1.16, *p* < 0.0001). Increased miR-26a-5p was predicted to inhibit 4 molecules indirectly. Only reduced expression of GSK3β, however, was validated in GEO-GSE17054 with a 0.33-fold change in relative expression (log_2_ value of − 1.60, *p* = 0.002). Reduced levels of miR-339-3p in AML-derived MSC exosomes was predicted to increase the expression of 14 potential targets through a loss of direct inhibition and 3 molecules were validated in GEO-GSE30029. Expression of KRBA2 increased 5.0-fold (log_2_ value of 2.32, *p* < 0.0001), RRBP1 increased 3.9-fold (log_2_ value of 1.96, *p* < 0.0001), and HIST2H2BE increased 5.1-fold (log_2_ value of 2.34, *p* < 0.0001) in AML samples compared to controls. miR-425-5p was predicted to interact with 23 different targets as a direct inhibitor. While levels of gene expression were predicted to increase in leukemia cells for all identified potential targets of reduced levels of miR-425-5p, only expression of APOBEC3A was significantly different and was decreased (opposite direction than predicted) with a 0.2-fold change in expression (log_2_ value of − 2.33, *p* = 0.008). The molecules with significant differential gene expression in AML CD34-selected cells compared to controls that were predicted by the miR profiling are summarized in Table 8. See Supplemental Fig. 1 for specific gene expression values for each gene validated by the gene expression datasets.

**Table 8:** Validated gene expression data for targets of microRNA species elevated in AML-derived MSC exosomes or control MSC-derived exosomes

miR in AML-derived MSC exosomes vs control	Altered gene expression in AML vs control, predicted by miR targets
↑ miR-101-3p	EZH2 ↓
↑ miR-26a-5p	GSK3B↓
↓ miR-339-3p	KRBA2 ↑
	RRBP1 ↑
	HIST2H 2BE ↑
↓ miR-425-5p	–
↓ miR-23b-5p	–

The expression of the five target genes that were validated in the publicly available gene expression datasets were then assessed using quantitative RT-PCR in primary AML-derived CD34-selected cells from five patient samples, three of which were derived from the same patient sample as the MSCs for the exosomal microRNA profiling, and healthy control CD34-selected bone marrow cells. The overall expression of all five genes was consistent with the data from gene expression profiling datasets. Specifically, the mean expression of EZH2 (1.62 fold decreased, SD 0.36) and GSK3 (1.60 fold decreased, SD 0.35) were reduced in all 5 AML-derived samples compared with controls and the mean expression of KRBA2 (2.34 fold increased, SD 0.75), RRBP1 (3.16 fold increased, SD 1.39), HIST2H2BE (3.04 fold increased, SD 0.90) were all increased (see supplemental Fig. 2).

## Discussion

Gene regulatory networks in AML may be influenced by microRNAs contained in MSC-derived exosomes. In our small study, profiling of miRs in exosomes from AML-derived MSCs compared to normal MSCs allowed us to identify candidate miRs with potential relevance in AML. By validating the predicted changes in gene expression of known targets of the five candidate miRs in datasets from previously studied AML cells relative to normal hematopoietic progenitors, our preliminary study highlights a novel approach that can be used to identify molecular pathways implicated in microenvironment-induced leukemogenesis. Some of the targets have been previously associated with AML while others have not been previously implicated. Further investigation of the molecular pathways identified in our study will be needed to gain insight regarding MSC-derived exosomal miRs in leukemogenesis, disease evolution, or potential new treatment strategies.

The role of the bone marrow microenvironment in leukemia has been studied previously (reviewed in [14]) and examples of factors produced by cells in the microenvironment have emerged. For example, studies suggest that the microenvironment may produce factors that can induce tumor cell quiescence. In one report, production of osteopontin by osteoblasts facilitated adhesion of acute lymphoblastic leukemia cells to specific anatomic locations in the marrow that induced leukemia cell dormancy and persistence of minimal residual disease following cytotoxic therapy in a xenografted mouse model [15]. Moreover, AML cells overexpressing Axl were able to induce the expression of Gas6 in bone marrow stromal cells which acts on the Axl-expressing AML cells to induce proliferation, survival and chemoresistance in the leukemia cells [15]. Downregulation of connective tissue growth factor in MSCs allowed Ben-Battala et al. [16, 17] to create human extramedullary bone marrow in a xenogeniec mouse transplant model, which demonstrated marked engraftment of leptin receptor-enriched leukemia cells due to increased leptin production by MSCs. In addition, previous studies by our group have demonstrated abnormalities in MSCs derived from patients with AML compared to controls, including reduced expression of genes associated with the maintenance of normal hematopoiesis and reduced capacity to support colony-formation of hematopoietic progenitors. Taken together, changes in the microenvironment of the marrow may create specific conditions that inhibit normal hematopoiesis and increase the relative competitiveness of leukemic cells to occupy specialized stem cell niches. The extent to which changes in MSCs contribute to leukemogenesis or alter gene regulatory networks in leukemia, however, remains less clear.

The release of factors by MSCs, including the release of microvesicles, may allow MSCs to alter the biology of malignant cells occupying niches within the marrow microenvironment. For instance, exosomes from MSCs in patients with multiple myeloma contributed to tumor growth in mice injected with cells from myeloma cell lines whereas mice treated with exosomes from normal MSCs demonstrated inhibition of tumor growth [11]. The impact of exosome-derived microRNAs released from the microenvironment is further illustrated by related work on MSC-derived exosomes in patients with stroke. MicroRNA 133b (miR133b) was transferred to astrocytes and neurons from MSC-derived exosomes that were infused following middle cerebral artery occlusion in rats and accelerated functional recovery and neurite remodeling [18]. The identification of microRNA species and/or proteins that can be transferred from MSCs to leukemia cells or to hematopoietic stem cells in humans represents a novel and promising concept for the development of new therapeutic strategies to treat AML.

While the expression of microRNAs was not different in the parent MSCs in our study, differential packaging of microRNAs into exosomes has been reported previously [19, 20] and may account for the different levels observed in our study. MicroRNAs that are packaged in exosomes of AML-derived MSCs can provide clues to potential disruption of several gene regulatory pathways that may be involved in leukemogenesis. We validated reduced expression of EZH2 in AML cells compared to control CD34-selected cells in one public dataset and confirmed that EZH2 can be inhibited by miR-101-3p which was increased in MSC-derived exosomes in patients with AML. While EZH2 mutations have been described in approximately 2% of de novo acute leukemia [21], genomic loss of EZH2 can lead to epigenetic changes and overexpression of the HOX genes in myelodysplastic syndrome [22, 23]. While increased transformation to acute leukemia from myelodysplasia in EZH2-deleted cases has not been demonstrated, pre-leukemic changes associated with MDS coupled with other changes may predispose patients to the development of AML. In some cases of secondary AML related to prior chemotherapy, EZH2 expression is increased. The precise role of EZH2 in AML and its evolution and response to treatment continues to be studied. Reduced GSK-3β expression has been previously associated with the development of acute leukemia in mice where removal of GSK-3β expression lead to aggressive AML through altered Wnt/Akt/mTOR signalling [24]. Our finding of reduced GSK-3β expression in AML is consistent with greater inhibition by miR-26a-5p in MSC-derived exosomes compared to controls, however, studies in human leukemia are needed to gain more mechanistic insight. Phosphorylation of GSK-3β in AML, however, can activate the Akt pathway and is associated with poorer overall survival in AML patients [25]. Expression of KRBA2, RRBP1 and HIST2H 2BE were increased in AML-derived CD34-selected cells in one dataset compared to controls but these three genes have not been previously associated with AML to the best of our knowledge. While we also observed gene expression of the five target genes by RT-qPCR in AML-derived samples was consistent with publicly available datasets from genome-wide gene expression profiling, the role of these genes in human AML warrants further validation and understanding. Further insight regarding the impact of microenvironment-derived microRNAs on leukemia cells and normal HSCs will allow us to understand how the marrow stroma can communicate with hematopoietic progenitors and their leukemic counterparts. Knowing how the microenvironment favors leukemic cell survival and inhibits normal hematopoiesis may allow us to target the microenvironment in AML using novel interventions to facilitate more competitive repopulation of the marrow with normal hematopoiesis and overcome treatment resistance in leukemia. There is no reported role of miR-101-3p in leukemia or myelodysplasia to the best of our knowledge. Indeed, one recent study examined whether miR-101-3p was differentially regulated in MDS compared to controls and did not find any significant difference [26].

Future studies that enrol greater numbers of patients will be needed to gain insight regarding any potential therapeutic targets arising from our work and also to understand if differentially packaged microRNAs in MSC-derived exosomes can provide a potential biomarker panel to screen MSCs in patients treated for AML to assess whether the microenvironment has normalized. We hypothesize that normalization of the microenvironment is required for sustained remission of AML. Persistence of MSC-derived signalling abnormalities may perpetuate inhibition of normal hematopoiesis and the continued competitive advantage of leukemia cells for stem cell niches in the marrow.

Limitations of our study are worth mentioning. Preparing MSC-derived exosomes and RNA sequencing is laborious and a larger sample size is needed to corroborate our findings in separate populations, including various subtypes of AML with different molecular and cytogenetic abnormalities, in older patients with AML, patients with secondary AML, and patients with refractory AML. Moreover, the effect of treatment and transplantation on MSC-derived exosomes remains to be studied. There are variations in technical approaches to the isolation of exosomes although the use of the approach applied in our study has been associated with high purity compared to other methods in a recent study [27]. While profiling small numbers of samples may be a drawback, the use of RNA sequencing increases confidence regarding the fidelity of nucleic acid sequence and identification of miRs. Validation in public datasets was limited by the numbers of patients and controls, and AML subtype frequencies. Further validation work is needed to understand how exosomal microRNA gene regulatory influences are offset by other regulatory controls in target cells.

In summary, MSC-derived exosomal microRNA represents a potential mechanism for influencing gene regulatory networks in AML. Candidate miRs were identified with corroborating changes in gene expression of known targets in AML samples. Our work provides justification for pursuing additional studies to understand better the microenvironment-induced changes in AML.

## Electronic Supplementary Material

Below is the link to the electronic supplementary material.

Supplementary material 1 (DOCX 24 KB)

Supplementary material 2 (DOCX 104 KB)

Supplementary material 3 (DOCX 27 KB)

Supplementary material 4 (DOCX 94 KB)
