# Supplementary material for: Micro-RNA Profiling of Exosomes from Marrow-Derived Mesenchymal Stromal Cells in Patients with Acute Myeloid Leukemia: Implications in Leukemogenesis
Source: Stem Cell Rev. 2017 Sep 16;13(6):817–25. doi: 10.1007/s12015-017-9762-0 (PMC5730624; doi:10.1007/s12015-017-9762-0)
Supplement: Supplementary file 1 — Supplementary material 1 (DOCX 24 KB) [file 12015_2017_9762_MOESM1_ESM.docx]

**Supplemental Table 1: primers used RT-PCR of 5 target genes in CD34-selected AML cells and CD34-selected bone marrow cells from healthy controls.**

| **Primer Name** | **Sequence** |
| --- | --- |
| EZH2-F | CCACAGTGTTACCAGCATTTG |
| EZH2-R | ACTGTTATTGGGAAGCCGTC |
| GSK3B-F | GGTCTATCTTAATCTGGTGCTGG |
| GSK3B-R | TGGATATAGGCTAAACTTCGGAAC |
| KRBA2-F | AACCCAGCACCTCACAAG |
| KRBA2-R | GCAGCCTTCAATCTTTTCCTC |
| RRBP1-F | TTGAAAGTTCGGACCAGGTG |
| RRBP1-R | CGAGCTGAGATTGAGATTCTAGG |
| HIST2H2BE-F | GCGTTTGTACTGTGTCTTACC |
| HIST2H2BE-R | ACACGTAGATGGAGTAGCTCTC |
