# Supplementary material for: Micro-RNA Profiling of Exosomes from Marrow-Derived Mesenchymal Stromal Cells in Patients with Acute Myeloid Leukemia: Implications in Leukemogenesis
Source: Stem Cell Rev. 2017 Sep 16;13(6):817–25. doi: 10.1007/s12015-017-9762-0 (PMC5730624; doi:10.1007/s12015-017-9762-0)
Supplement: Supplementary file 2 — Supplementary material 2 (DOCX 104 KB) [file 12015_2017_9762_MOESM2_ESM.docx]

Supplemental Figure 2: Gene expression data by RT-qPCR of genes in AML-derived CD34+ cells compared to healthy bone-marrow-derived CD34+ cells.
