# Supplementary material for: Micro-RNA Profiling of Exosomes from Marrow-Derived Mesenchymal Stromal Cells in Patients with Acute Myeloid Leukemia: Implications in Leukemogenesis
Source: Stem Cell Rev. 2017 Sep 16;13(6):817–25. doi: 10.1007/s12015-017-9762-0 (PMC5730624; doi:10.1007/s12015-017-9762-0)
Supplement: Supplementary file 3 — Supplementary material 3 (DOCX 27 KB) [file 12015_2017_9762_MOESM3_ESM.docx]

**Supplemental Table 2.** Most abundant (top 30) human microRNA (miR) species in exosomes from AML and control-derived MSCs. Copy number of miR species represents mean value ± standard deviation, normalized to picograms of total miR. No significant difference in mean copy numbers was observed between groups.

| microRNA | AML MSC exosomes | control MSC exosomes |
| --- | --- | --- |
| miR-122-5p | 3680 ±1980 | 3413 ± 838 |
| miR-100-5p | 3159 ± 403 | 2746 ± 900 |
| miR-21-5p | 2827 ± 546 | 2956 ± 479 |
| let-7c-5p | 1035 ± 553 | 720 ± 251 |
| let-7b-5p | 784 ± 354 | 613 ± 135 |
| let-7a-5p | 742 ± 457 | 431 ± 230 |
| miR-27a-3p | 607 ± 73 | 734 ± 57 |
| miR-27b-3p | 592 ± 78 | 706 ± 125 |
| miR-10a-5p | 434 ± 108 | 308 ± 103 |
| miR-221-3p | 414 ± 117 | 424 ± 146 |
| miR-99a-5p | 375 ± 27 | 286 ± 146 |
| miR-423-5p | 371 ± 220 | 415 ± 26 |
| miR-3184-3p | 371 ± 220 | 415 ± 148 |
| miR-615-3p | 330 ± 244 | 341 ± 39 |
| miR-199a-3p | 307 ± 65 | 338 ± 39 |
| miR-199b-3p | 307 ± 65 | 338 ± 60 |
| miR-22-3p | 298 ± 67 | 376 ± 259 |
| miR-24-3p | 286 ± 119 | 319 ± 54 |
| miR-29a-3p | 214 ± 132 | 171 ± 83 |
| let-7i-5p | 203 ± 91 | 258 ± 39 |
| miR-125b-5p | 203 ± 32 | 184 ± 52 |
| miR-143-3p | 198 ± 112 | 243 ± 30 |
| miR-10b-5p | 178 ± 39 | 202 ± 53 |
| miR-320b | 171 ± 43 | 195 ± 49 |
| miR-320a | 168 ± 43 | 192 ± 43 |
| miR-3591-3p | 156 ± 63 | 183 ± 61 |
| miR-181a-5p | 151 ± 10 | 155 ± 30 |
| miR-1290 | 143 ± 26 | 313 ± 25 |
| miR-193a-5p | 139 ± 65 | 158 ± 14 |
| miR-125b-1-3p | 123 ± 23 | 153 ± 35 |
