# Supplementary material for: Micro-RNA Profiling of Exosomes from Marrow-Derived Mesenchymal Stromal Cells in Patients with Acute Myeloid Leukemia: Implications in Leukemogenesis
Source: Stem Cell Rev. 2017 Sep 16;13(6):817–25. doi: 10.1007/s12015-017-9762-0 (PMC5730624; doi:10.1007/s12015-017-9762-0)

**Supplemental Figure 1.** Gene expression data in AML-derived CD34-selected cells (blue) and healthy control CD34-selected cells (red) from bone marrow samples for (A) EZH2; (B) GSK3B; (C) KRBA2; (D) RRBP1 (data shown for 3 distinct gene identifiers), and (E) HIST2H 2BE. Data was extracted from the database by de Jonge et al. (2011) ^[3]^

**A ) EZH2** – ILMN_1708105


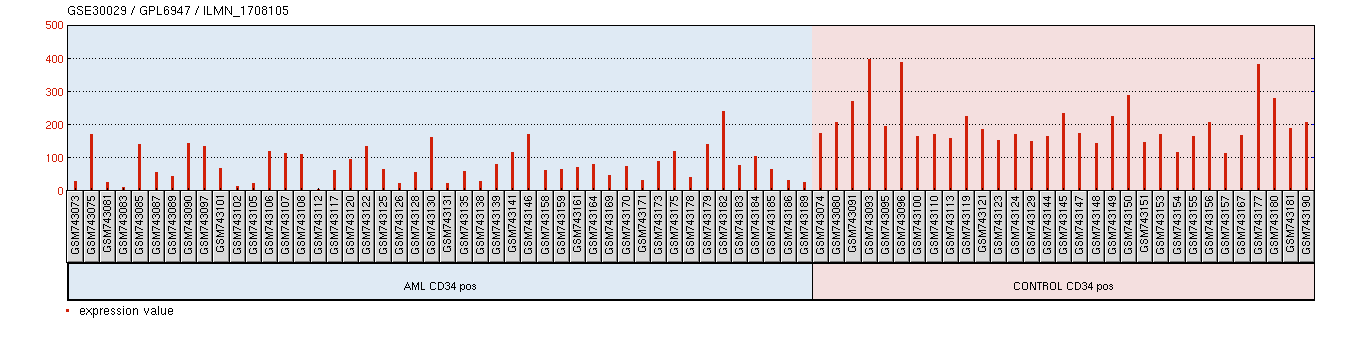


**(B) GSK3B** – ILMN_ 1779376


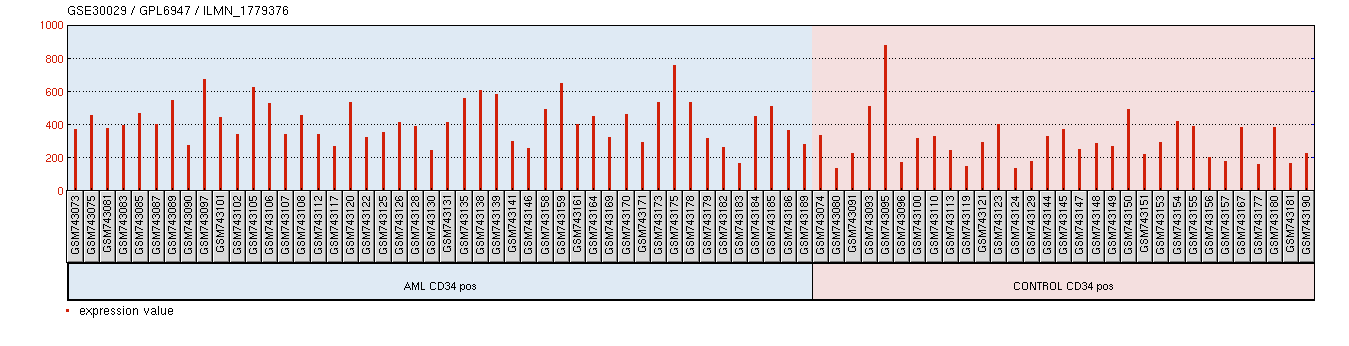


**C) KRBA2** – ILMN_ 1685202


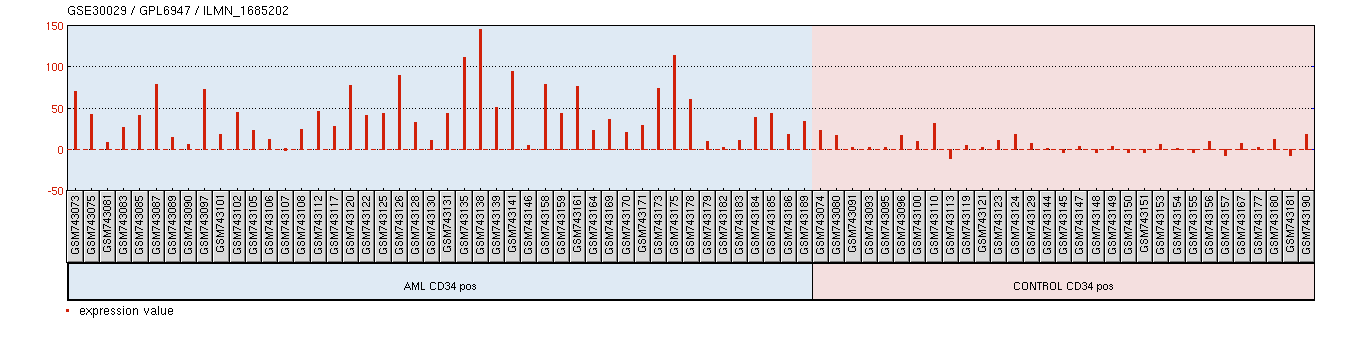


**(D) RRBP1**– ILMN_ 2360784


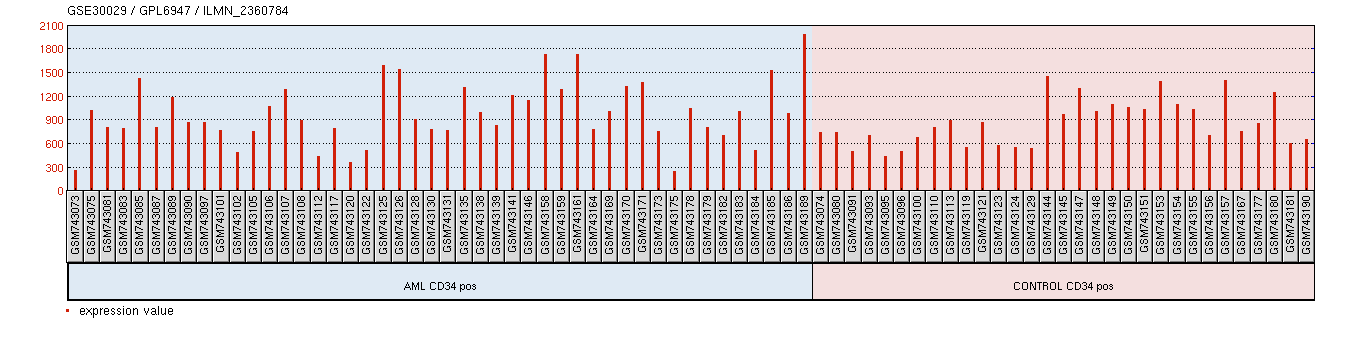


ILMN_2268381


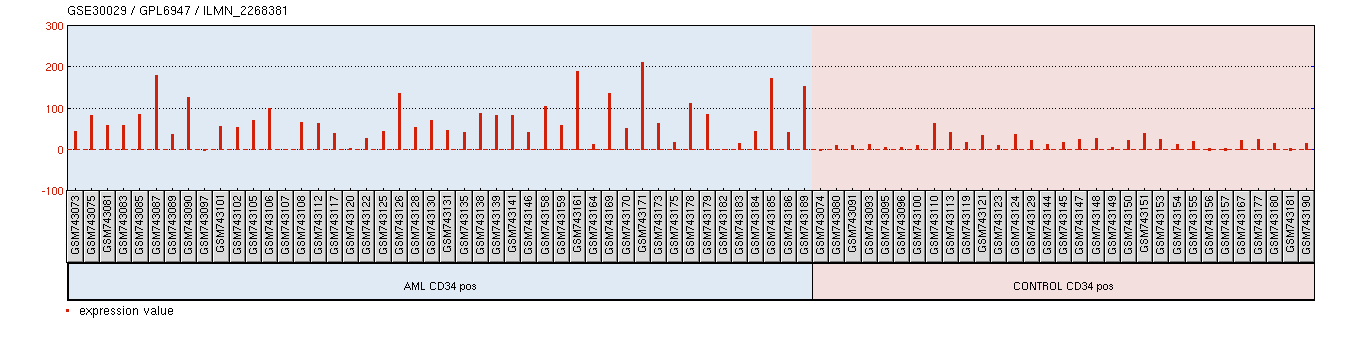


ILMN_1803810
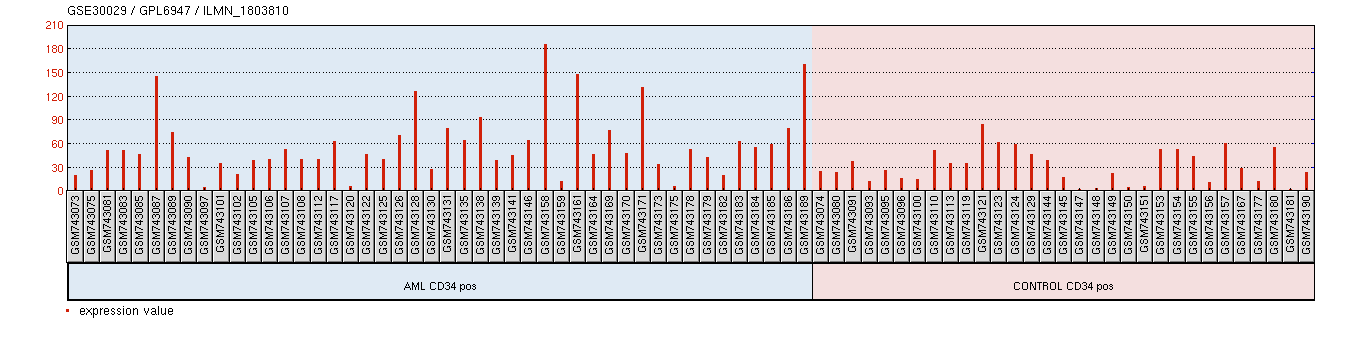


**E) HIST2H2BE** – ILMN_1732071


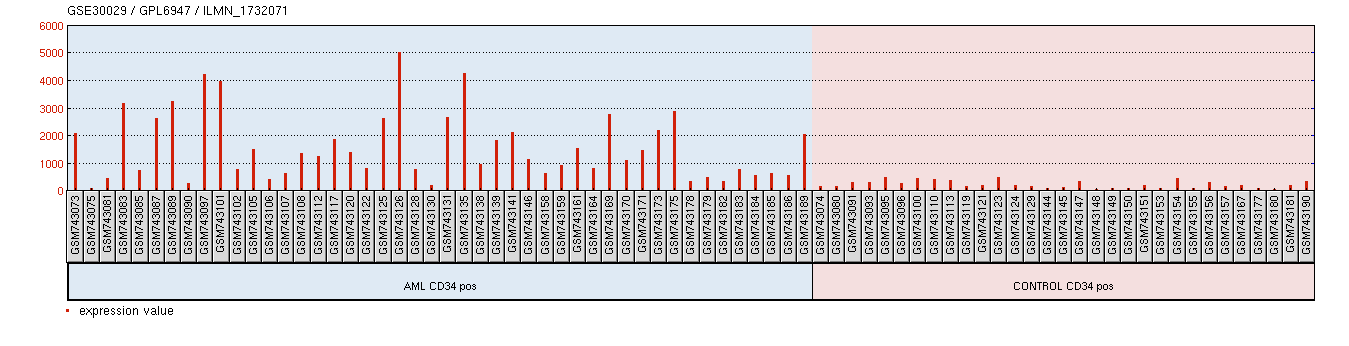

Supplement: Supplementary file 4 — Supplementary material 4 (DOCX 94 KB) [file 12015_2017_9762_MOESM4_ESM.docx]
